# RKDOSCNV: A Local Kernel Density-Based Approach to the Detection of Copy Number Variations by Using Next-Generation Sequencing Data

**DOI:** 10.3389/fgene.2020.569227

**Published:** 2020-11-04

**Authors:** Guojun Liu, Junying Zhang, Xiguo Yuan, Chao Wei

**Affiliations:** School of Computer Science and Technology, Xidian University, Xi'an, China

**Keywords:** copy number variation, next-generation sequencing, kernel density estimation, split read, biological meanings

## Abstract

Copy number variations (CNVs) are significant causes of many human cancers and genetic diseases. The detection of CNVs has become a common method by which to analyze human diseases using next-generation sequencing (NGS) data. However, effective detection of insignificant CNVs is still a challenging task. In this study, we propose a new detection method, RKDOSCNV, to meet the need. RKDOSCNV uses kernel density estimation method to evaluate the local kernel density distribution of each read depth segment (RDS) based on an expanded nearest neighbor (k-nearest neighbors, reverse nearest neighbors, and shared nearest neighbors of each RDS) data set, and assigns a relative kernel density outlier score (RKDOS) for each RDS. According to the RKDOS profile, RKDOSCNV predicts the candidate CNVs by choosing a reasonable threshold, which it uses split read approach to correct the boundaries of candidate CNVs. The performance of RKDOSCNV is assessed by comparing it with several current popular methods via experiments with simulated and real data at different tumor purity levels. The experimental results verify that the performance of RKDOSCNV is superior to that of several other methods. In summary, RKDOSCNV is a simple and effective method for the detection of CNVs from whole genome sequencing (WGS) data, especially for samples with low tumor purity.

## Introduction

With the rapid development of next-generation sequencing (NGS) technology, many sequencing data sets that are used to detect and characterize human genome variation have been produced (Medvedev et al., 2009). Copy number variation (CNV) is one of the important forms of genome structural variation (Freeman et al., 2006). It has been reported that many human cancers and diseases are caused directly or indirectly by CNVs (Zhao et al., 2013). It is therefore necessary for humans to accurately detect CNVs using NGS data to effectively discover disease-causing genes and develop targeted drugs (Yuan et al., 2018). The workflow of the general CNV detection method includes the following steps: (1) comparing reads to a reference genome (Metzker, 2010) and generating a SAM file with BWA (Li and Durbin, 2010); (2) obtaining a read count (RC) profile using SAMtools (Li et al., 2009); (3) binning the RC profile (Chiang et al., 2009) and obtaining the read depth (RD) profile; (4) preprocessing the RD profile [eliminating GC bias, removing mappability bias (Dohm et al., 2008), and denoising the data (Cai et al., 2018)]; (5) modeling and forecasting CNVs. In recent years, many CNV detection methods have been developed based on this process. According to the number of entered samples, the CNV detection methods can be divided into three categories, namely those that use multiple samples, matched case-control samples, and a single sample, respectively.

Many detection methods have been developed based on multiple samples as inputs, such as cn.MOPS (Povysil et al., 2017), RDXplorer (Yoon et al., 2009), CoNIFER (Krumm et al., 2012), XHMM (Fromer et al., 2012), and CODEX (Jiang et al., 2015). The cn.MOPS method assumes that the RD at each location is subject to a hybrid Poisson model that is used to predict CNVs. RDXplorer detects CNVs using an event-wise testing method, namely significance testing based on preprocessed RDs of coverage. CoNIFER uses singular value decomposition to detect CNVs and discover genotype copy number polymorphic loci. XHMM is a statistical tool that uses principal component analysis to preprocess RD profiles, and establishes a hidden Markov model to announce CNVs. CODEX builds a Poisson latent factor model to normalize RDs, and uses a Poisson likelihood-based recursive segmentation procedure to declare CNVs. Multiple sample-based methods are suitable for the detection of the driver genes of cancer in the same diseased group, which is conducive to the development of some targeted drugs for clinical treatment. Via the preceding analysis, it is clear that most CNV detection methods based on multiple samples use a distribution model to fit the RD signals. However, in practical applications, RD signals do not obey the assumed distribution due to data noise, sequencing errors, and sample contamination, which leads to inaccurate test results. In daily life, doctors often encounter patients with different diseases who require different treatment plans. Therefore, the development of single sample-based methods is imperative.

Some popular methods based on matched case-control samples have been developed in recent years, and include XCAVATOR (Magi et al., 2017), SeqCNV (Chen et al., 2017), BIC-seq (Xi et al., 2010), CNAnorm (Gusnanto et al., 2012), and CNAseg (Ivakhno et al., 2010). XCAVATOR uses a two-step procedure in which RC biases are removed to identify the absolute copy number and a shifting-level model method is used to predict CNVs. SeqCNV comprises a maximum penalized likelihood estimation method to calculate the copy number ratio and detect CNVs. BIC-seq adopts the minimization of the Bayesian information criterion method to identify CNVs based on RD information. CNAnorm calibrates contamination with normal cells and assesses the ploidy to calculate the copy numbers of detected areas. CNAseg adopts flowcell-to-flowcell variability in case and control samples to reduce the false positive rate and identify CNVs. The matched case-control sample-based methods are suitable for detection in individual patients, and can be used to identify germline and somatic CNVs. These methods can detect disease-causing genes associated with cancer and identify the difference between the normal and abnormal tissues of a single patient, and can effectively identify somatic CNVs using targeted NGS data, which is very important especially in clinical examination and cancer research. However, these methods also have some limitations and a control sample of a patient is required, the collection and production of which are relatively expensive. Via the preceding analysis, it is evident that the paired sample-based methods are suitable for the detection of disease in niche people or for conducting experimental research that requires specific clinical needs.

Due to the needs of practical applications, single sample-based CNV detection methods emerged. In contrast to the two other types of methods, these methods only require a sample as the input, which reduces the cost of patient testing. For example, as compared with the paired sample-based methods, the cost of patient testing is reduced by about half because the provision of a control sample is not required. In recent years, many single sample-based methods have been developed, most of which use depth of coverage (DOC) information to establish a model and forecast CNVs; the basic principle is that the RC of each location of a reference genome is proportional to the copy number of each location (Yoon et al., 2009). In theory, DOC-based methods can detect CNVs of any size; thus, the vast majority of existing methods have been developed based on DOC technology, and include ReadDepth (Miller et al., 2011), CNVnator (Abyzov et al., 2011), GROM-RD (Smith et al., 2015), iCopyDAV (Dharanipragada et al., 2018), FREEC (Boeva et al., 2011), and CNV_IFTV (Yuan et al., 2019). ReadDepth employs preprocessed RD information to fit a negative binomial distribution and circular binary segmentation to forecast CNVs; it is suitable for the detection of high-purity tumor samples, though its accuracy is relatively low. CNVnator uses the mean-shift method with GC content correction and multiple-bandwidth partitioning to discover CNVs; it is not suitable for testing low-purity samples or the investigation of short CNVs, and, while it can achieve a high recall rate, it has low precision. GROM-RD corrects repeat deviations and adopts a two-pipeline masking approach to discover CNVs in duplicate and complex areas; however, its performance is limited to the detection of low-purity tumor samples, and the false positive rate of test results is relatively high. iCopyDAV is an integrated software platform composed of the detection, annotation, and visualization of CNVs, and is suitable for testing samples with high purity and medium coverage. FREEC preprocesses the RD with GC content and uses GC content profiles for segmentation, which is used to allocate copy numbers for each area. Its performance is more balanced, and it achieves high recall and precision. CNV_IFTV calculates the anomaly score for each area with the isolation forest method and employs a total variation model to smooth the anomaly score profile, based on which a gamma distribution is established to predict CNVs. It achieves a better tradeoff between recall and precision as compared to the other previously mentioned methods, but it has a higher time complexity. Via the analysis of these methods, it is evident that the development of some new methods with complete functions based on a single sample is necessary. The existing methods mainly have the following limitations. (1) The general methods assume that the RD information follows a certain distribution, such as a negative binomial distribution or a mixed Poisson distribution. In actual experiments, it is found that the distribution of RD signals deviates from the assumption, which is caused by sequencing errors, GC-content bias, mappability bias, and experimental noise. (2) Most methods only use RD information to build models and predict CNVs. In theory, these methods can detect CNVs of any size and type, but cannot accurately detect the boundaries of variation regions. (3) It is very difficult for most methods to detect the hemizygous loss regions, the copy numbers of which are one. The regions can easily be regarded as normal areas with copy numbers of two. Therefore, it is necessary to adopt reasonable strategies and reduce the impact on the test results.

Based on the previously discussed considerations, a novel method, called RKDOSCNV (local kernel density estimation-based approach for CNV detection), is developed in this study to predict CNVs from single tumor samples via the use of whole genome sequencing (WGS) data. A kernel density estimation (KDE) method is adopted to evaluate the distribution of the local kernel density (LKD) for each read depth segment (RDS) based on the extended nearest-neighbor data set, which is composed of the k-nearest neighbors (KNNs), reverse nearest neighbors (RNNs), and shared nearest neighbors (SNNs) of each RDS (Tang and He, 2017). After the calculation of the LKD of RDSs, the relative kernel density outlier score (RKDOS) is evaluated for each RDS, which can indirectly reflect the degree of deviation of each RDS as compared to its three types of nearest neighbors. By choosing a suitable threshold θ, the RKDOS of each RDS is compared with θ. For example, if the RKDOS of an RDS is greater than θ, it is considered as a candidate CNV. Based on the prediction results, the boundaries of candidate CNVs are more precisely refined using split read (SR) approach. The performance of the proposed RKDOSCNV is estimated based on simulated data sets and compared with the performances of several popular methods. To further verify the validity of RKDOSCNV, it is used to detect real tumor samples, and it is found that some CNVs are associated with cancers and diseases, thus proving the effectiveness of the method.

## Method and Materials

### Overview of RKDOSCNV

**Figure 1:**
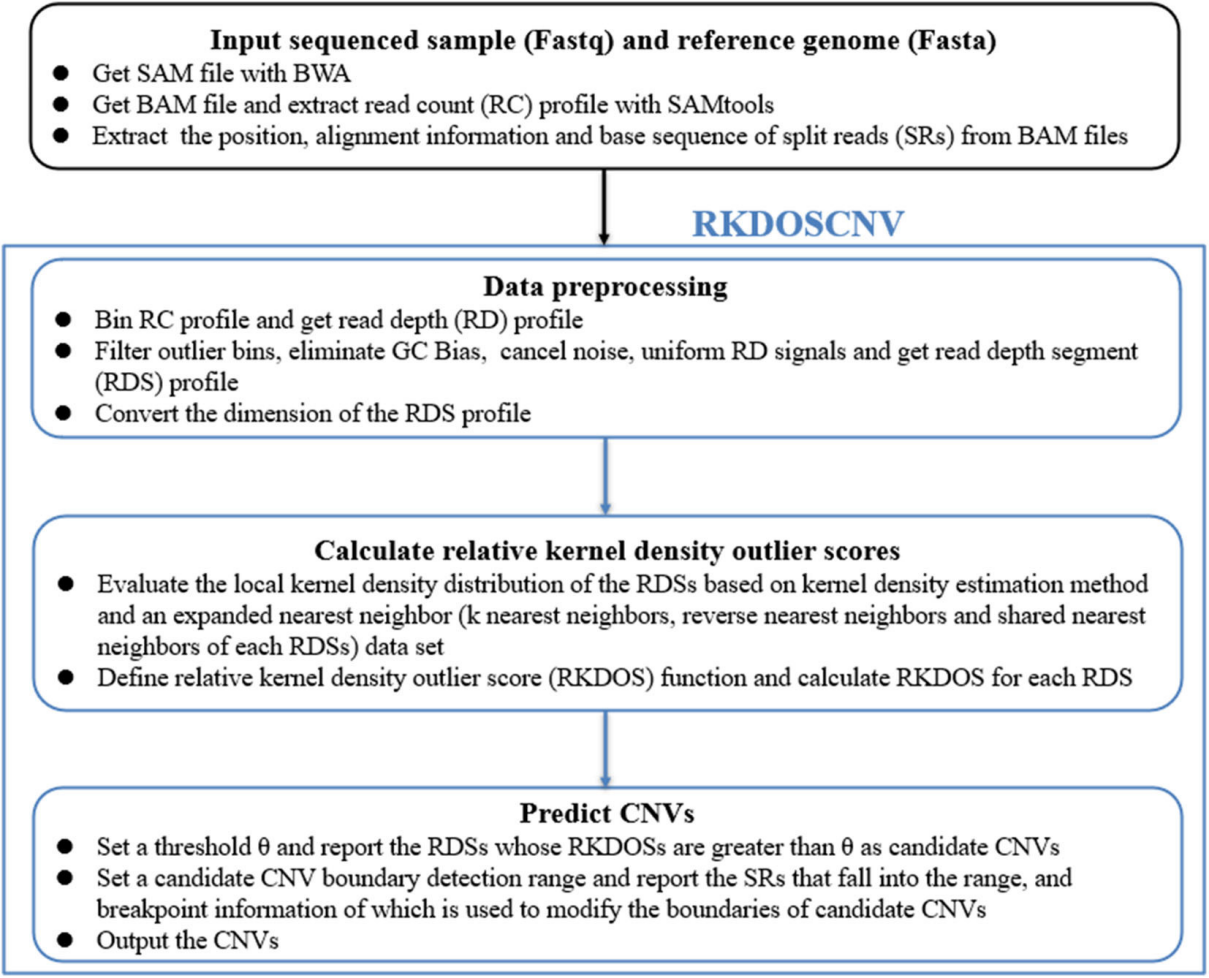
Overview of the workflow of RKDOSCNV. It is mainly composed of four parts, namely input file preparation, data preprocessing, relative kernel density outlier score calculation, and CNV prediction.

RKDOSCNV is developed based on the DOC and SR methods, and is applied to detect single tumor samples without the provision of a matched control sample. Figure 1 describes in detail the overall workflow of RKDOSCNV, which is composed of the following four steps. First, the sequenced sample (Fastq) and reference genome (Fasta) are offered as inputs. Many of the reads that come from the Fastq file are compared to the reference genome (e.g., HG19), which can generate a SAM file using BWA (Li and Durbin, 2010). The SAM file can be converted to a BAM file, and the RC profile is extracted from the SAM file with SAMtools (Li et al., 2009). The positions, alignment information, and base sequences of SRs are further collected from BAM files. The second step mainly includes defining the bins, filtering anomalous bins, calibrating the GC bias, noise reduction, balancing RD signals, and converting the dimensions of RD data. The third step, RKDOSCNV calculates the RKDOS for each RDS based on the extended nearest-neighbor data set (Tang and He, 2017). Finally, RKDOSCNV reports candidate CNV regions whose RKDOSs are > θ, which is a set threshold, and applies the SR approach to further refine the boundaries of the candidate CNV regions. The RKDOSCNV software is developed in the R and Python languages. It can be downloaded from https://github.com/gj-123/RKDOSCNV/releases, and is easy to install and use after reading the user manual.

### Data Preprocessing

The sequenced sample is compared to the reference genome by BWA, which obtains the SAM file from which the RC profile is extracted with SAMtools. The reference genome is composed of many “A,” “T,” “G,” “C,” and “N,” of which “N” represents the positions of the reference genome that are missing in the sequencing process. When reads compares to positions of “N,” which will cause the RCs to be equal to zero at these positions (Yuan et al., 2018). To obtain a reasonable RC profile, the following strategies are adopted to solve this problem. A bin is defined, which is a continuous and non-overlapping sliding window. In this study, the bin size was set to 2000 bp. If a bin contains positions of “N,” it will be filtered as an abnormal bin. An RD is defined as a numerical value that can be determined by calculating the RC mean of a bin. Based on this processing, the GC bias is caused by the PCR amplification that is calibrated by Equation (1) (Yoon et al., 2009):

(1) $$\begin{array}{l} RD_{i}^{\prime }=\frac{\bar{RD}\cdot RD_{i}}{RD_{gc}}, \end{array}$$

where $RD_{i}^{\prime }$ represents the RD of the *i*-th bin after correction, $\bar{RD}$ represents the mean of all RDs, *RD*_*i*_ represents the RD of the *i*-th bin, and *RD*_*gc*_ denotes the mean of RDs that have the same GC content.

The noise of RDs severely affects the accuracy of CNV detection; the noisy data is directly used to detect CNVs, which results in inaccurate detection results. Therefore, the elimination of noise is a critical step in the CNV detection process. In this study, a total variation method is adopted, which can reduce noise, segment, and smooth one-dimensional discrete signals based on the regularized total variation (Condat, 2013) and least-squares approaches (Duan et al., 2013). With the RDS profile, the RDS signals are asymmetric because the copy number gains and losses are asymmetric as compared with the normal copy number. If the model is based on asymmetric RD signals, some insignificant CNVs will be easily overlooked. Here, Equation (2) is used to adjust the symmetry of RD signals (Yuan et al., 2018):

(2) $$\begin{array}{l} RDS_{i}^{\prime }=\left|\frac{RDS_{\text{min}}}{RDS_{\text{max}}}\right|\cdot \left(RDS_{i}-RDS_{M}\right), \end{array}$$

where $RDS_{i}^{\prime }$ denotes the RD of the *i*-th segment after equalization, *RDS*_min_ denotes the minimum value of the RDSs, *RDS*_max_ denotes the maximum value of the RDSs, *RDS*_*i*_ denotes the RD of the *i*-th segment, and *RDS*_*M*_ denotes the mode of the RDSs. Based on the balanced RDS profile, the one-dimensional RDS profile is converted into a two-dimensional profile *RDS*_*s*_, which are described in detail by Equations (3–5), respectively.

(3) $$\begin{array}{l} RDS_{x}=\frac{RDS_{i}}{\bar{RDS}} \end{array}$$

(4) $$\begin{array}{l} RDS_{y}=\\ \begin{array}{l} \{\begin{array}{c} \frac{\overset{i+m}{\underset{j=i+1}{\sum }}|RDS_{x}\left[i\right]-RDS_{x}\left[j\right]|}{m}i=1\\ \frac{\overset{i-1}{\underset{j=1}{\sum }}|RDS_{x}\left[i\right]-RDS_{x}\left[j\right]|+\overset{i+m}{\underset{j=i+1}{\sum }}|RDS_{x}\left[i\right]-RDS_{x}\left[j\right]|}{i+m-1}1<i\leq m\\ \frac{\overset{i-1}{\underset{j=i-m}{\sum }}|RDS_{x}\left[i\right]-RDS_{x}\left[j\right]|+\overset{i+m}{\underset{j=i+1}{\sum }}|RDS_{x}\left[i\right]-RDS_{x}\left[j\right]|}{2m}m<i\leq |RDS_{x}|-m\\ \frac{\overset{i-1}{\underset{j=i-m}{\sum }}|RDS_{x}\left[i\right]-RDS_{x}\left[j\right]|+\overset{|RDS_{x}|-1}{\underset{j=i+1}{\sum }}|RDS_{x}\left[i\right]-RDS_{x}\left[j\right]|}{|RDS_{x}|-i-1+m}|RDS_{x}|-m<i\leq |RDS_{x}|-1\\ \frac{\overset{i-1}{\underset{j=i-m}{\sum }}|RDS_{x}\left[i\right]-RDS_{x}\left[j\right]|}{m}i=|RDS_{x}| \end{array} \end{array} \end{array}$$

(5) $$\begin{array}{l} RDS_{s}=\left\{RDS_{x},RDS_{y}\right\} \end{array}$$

In Equation (3), *RDS*_*i*_ represents the RD of the *i*-th segment, $\bar{RDS}$ represents the mean of all the RDSs, and *RDS*_*x*_ represents the ratio between *RDS*_*i*_ and $\bar{RDS}$, which indirectly reflects the distribution of the copy number at each location. In Equation (4), |*RDS*_*x*_| represents the number of elements in the *RDS*_*x*_. *RDS*_*y*_ represents the difference between an RDS and the surrounding RDSs in a certain depth of exploration (m), which is helpful for the detection of insignificant CNVs in the local range. Here, the default value of m is set to 10. In Equation (5), *RDS*_*s*_ represents a two-dimensional data set that is composed of *RDS*_*x*_ and *RDS*_*y*_, and each element of it is treated as an object (*O*). The main purpose of this step is conducive to the capture of local insignificant CNVs, and provides an effective data set for the calculation of the RKDOSs presented in the subsequent section.

### Calculation of Relative Kernel Density Outlier Scores

Based on the *RDS*_*s*_ profile, RKDOSCNV uses the KDE approach to evaluate an LKD distribution, and assigns the RKDOS that can reflect the degree of isolation of an object as compared with its three types of nearest neighbors for each RDS (Tang and He, 2017). This is different from the traditional RD-based CNV detection methods, which generally build statistical models according to the approximate distribution of RDSs and choose an appropriate significance level to predict CNVs. Here, the LKD distribution function is defined using Equations (6–9).

(6) $$\begin{array}{l} f\left(O\right)=\frac{1}{n}\overset{n}{\underset{i=1}{\sum }}\frac{1}{\sigma^{d}}K\left(\frac{O-O_{i}}{\sigma }\right) \end{array}$$

(7) $$\begin{array}{l} K\left(\frac{O-O_{i}}{\sigma }\right)=\frac{1}{{\left(2\pi \right)}^{\frac{d}{2}}}e^{\left(-\frac{\|O-O_{i}{\|}^{2}}{2\sigma^{2}}\right)} \end{array}$$

In Equation (6), *O* represents any element in the *RDS*_*s*_, *O*_*i*_ represents the *i*-th element in the *RDS*_*s*_, σ represents the bandwidth of the kernel function, *d* represents the dimension of *RDS*_*s*_, and $K\left(\frac{O-O_{i}}{\sigma }\right)$ represents a multivariate Gaussian kernel function and is defined in Equation (7). $\|O-O_{i}{\|}^{2}$ represents the Euclidean distance between and *O*_*i*_.To accurately calculate the LKD of *O*, its three types of neighbor relations (KNNs, RNNs, and SNNs) are calculated, and are used as kernels in the Gaussian kernel function and represented with three sets (*RDS*_*s*−*knn*_, *RDS*_*s*−*rnn*_, and *RDS*_*s*−*snn*_), which are described in detail by Equations (8–11).

(8) $$\begin{array}{l} RDS_{s-knn}(O)=(RDS_{s-knn}[1],RDS_{s-knn}[2],\ldots ,\\ \;RDS_{s-knn}[k]) \end{array}$$

(9) $$\begin{array}{l} RDS_{s-rnn}\left(O\right)=\left(RDS_{s-rnn}\left[1\right],RDS_{s-rnn}\left[2\right],...,RDS_{s-rnn}\left[i\right]\right)\\ \begin{array}{l} \;or\emptyset \end{array} \end{array}$$

(10) $$\begin{array}{l} RDS_{s-snn}(O)=(RDS_{s-snn}[1],RDS_{s-snn}[2],\ldots ,\\ \;RDS_{s-snn}[j])or\emptyset \end{array}$$

(11) $$\begin{array}{l} RDS_{s-u}\left(O\right)=RDS_{s-knn}\left(O\right)\cup RDS_{s-rnn}\left(O\right)\cup RDS_{s-snn}\left(O\right)\\ \begin{array}{l} 1\leq i\leq k,1\leq j\leq k,i,j\in N \end{array} \end{array}$$

In Equation (8), *O* represents any object in the *RDS*_*s*_, *RDS*_*s*−*knn*_(*O*) represents the set of the KNNs of *O*. Here, the default value of k is set to 60. In Equation (9), *RDS*_*s*−*rnn*_(*O*) is a set that is composed of the RNNs of *O*, which are defined as the objects, the KNNs of which include object *O*. In practical applications, the RNNs of *O* may or may not exist. In Equation (10), *RDS*_*s*−*snn*_(*O*) represents the SNNs set of *O*, which is defined as the objects that have the same nearest neighbors as *O*. Similarly, the SNNs of *O* may or may not exist. In Equation (11), *RDS*_*s*−*u*_(*O*) represents the union of three types of nearest neighbors, based on which the proposed method can detect not only an isolated anomalous individual, but also a cluster of local insignificant anomalous individuals. The Equation (12) is used to calculate the LKD of *O*:

(12) $$\begin{array}{l} f\left(O\right)=\frac{1}{\left|RDS_{s-u}\left(O\right)\right|}\underset{O_{i}\in RDS_{s-u}\left(O\right)}{\sum }\frac{1}{\sigma^{d}}K\left(\frac{O_{i}-O}{\sigma }\right), \end{array}$$

where |*RDS*_*s*−*u*_(*O*)| represents the number of elements in the *RDS*_*s*−*u*_(*O*). Based on Equation (12), the calculation of the RKDOS for *O* is described by Equation (13):

(13) $$\begin{array}{l} RKDOS\left(O\right)=\frac{\underset{O_{i}\in RDS_{s-u}\left(O\right)}{\sum }f\left(O_{i}\right)}{|RDS_{s-u}\left(O\right)|f\left(O\right)}, \end{array}$$

where *RKDOS*(*O*) is defined as the ratio between the average of the LKD of the three types of nearest neighbors of *O* and the LKD of *O*.

### Declaring CNVs

**Figure 2:**
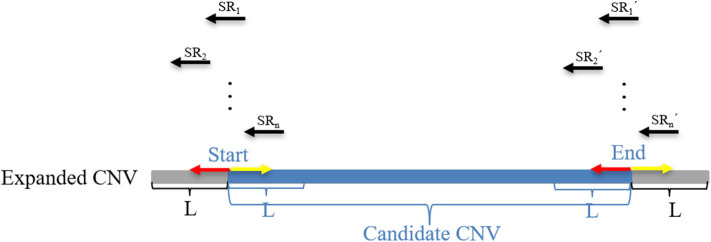
An example of the search SRs method. The black arrow represents the split reads (SRs) that fall into the areas of length 2*L* and are extracted from BAM files. The red arrow represents the forward search direction, and the yellow arrow represents the backward search direction. *L* represents the depth of the search. The blue area represents the candidate CNV area, and the gray area represents the expanded parts of the candidate CNV area, which constitute an expanded CNV region.

**Figure 3:**
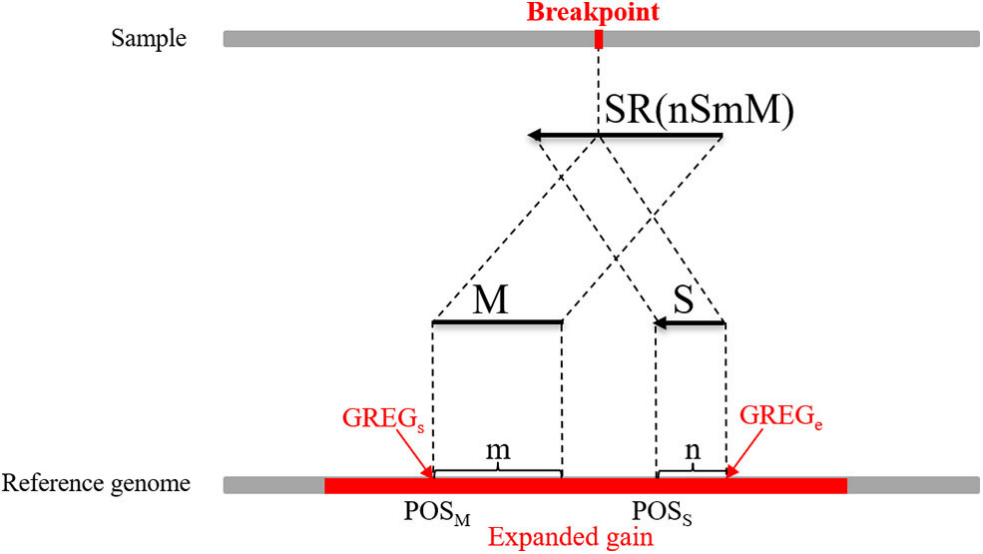
An example of the boundary detection method in the gain area. The split reads (SRs) come from a sequenced sample and contain breakpoint information, such as SR (*mSnM*). *M* represents the matched part of the SR, while *S* represents the unmatched part. Moreover, *m* represents the length of *M*, and *n* represents the length of *S*. The *M* and *S* of the SR are mapped on the reference genome. *POS*_*M*_ and *POS*_*S*_ represent the alignment positions of the first base at the left ends of *M* and *S*, respectively. The red area represents an expanded gain, the boundary of which is represented by *GREG*_*s*_ and *GREG*_*e*_.

**Figure 4:**
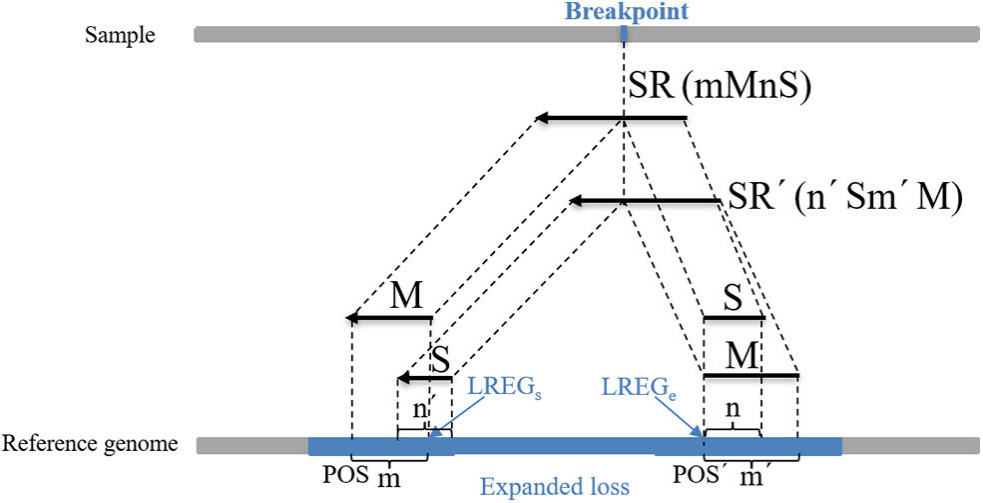
An example of the boundary detection method in the loss area. The split reads (SRs) come from a sequenced sample, and contain breakpoint information, such as SR (*mMnS*) and SR'(*n'Sm'M*). *M* represents the matched part of SR and SR', and *S* represents the unmatched part. Moreover, *m* and *m*' represent the length of *M* of SR and SR', and *n* and *n*' represent the length of *S*. This pair of SRs contains the same breakpoint, and their *M* and *S* are mapped on the reference genome. *POS* and *POS*' represent the alignment positions of the first base at the left ends of *M* of SR and SR', respectively. The blue area represents an expanded loss, the boundary of which is represented by *LREG*_*s*_ and *LREG*_*e*_.

Based on the RKDOS of each object, the degree of anomalies is progressively analyzed, and a threshold θ is chosen as the cutoff for those abnormal objects. The threshold θ is a constant that is determined by users according to their own application scenarios. In this work, the θ is set to 1.1. The basic judgment principle is as follows. (1) If the RKDOS of an object is > θ, it is considered as a candidate CNV area. (2) If the RKDOS of an object is ≤θ, it is considered as a candidate normal area. Structural variants (SVs) is an important manifestation of human chromosome variation, which includes the tandem amplification, interspersed amplification, deletion, insertion, and rearrangements (translocation and inversion) of DNA fragments (Stankiewicz and Lupski, 2010; Guan and Sung, 2016; Yuan et al., 2020). The SR-based SV and CNV detection methods can accurately detect the boundaries of the mutation areas, and reduce false positives and false negatives in the test results (Ye et al., 2009; Abyzov and Gerstein, 2011; Rausch et al., 2012; Layer et al., 2014). In this study, the SR approach is used to determine the locations of the breakpoints of candidate CNVs. Three features (the POS, CIGAR, and sequence) of SRs are extracted from BAM files (Li et al., 2009). The POS indicates the position of the leftmost first base of an SR sequence aligned to the reference genome. The CIGAR indicates the state of an SR aligned to the reference genome. It is a string, which is composed of numbers, *M* and *S*. Here, the type of CIGAR that only includes *M* and *S* is extracted, and is described by Equation (14):

(14) $$\begin{array}{l} CIGAR=\{\begin{array}{cc} mMnS & m,n\epsilon N,m+n=R_{l},\\ nSmM & ditto \end{array} \end{array}$$

where *M* represents an exact match, *S* represents no match, *m* indicates that the *m* bases of an SR completely match the reference genome, *n* indicates that the *n* bases of an SR cannot be completely matched to the reference genome, and *R*_*l*_ represents the length of an SR, which is generally set to 100 bp. Here, a CIGAR with a value of *n* > 10 is chosen. If the mismatch length of an SR is too small, it may be caused by sequencing errors. The sequence represents the base sequence of an SR. Most SRs are useless for boundary detection; therefore, conditions must be set to filter out effective SRs. Here, the search step size is set to the length of *n* bins (*L*). The starting point and endpoint indexes of a candidate CNV region that represent the leftmost position and rightmost position of a candidate CNV are respectively used as search centers according to which forward and backward searches are performed; This step forms two search ranges where those SRs are recorded. Figure 2 depicts an example of exploration process of SRs. The n SRs (from SR_1_ to SR_n_) and n SR's (from ${\text{SR}}_{1}^{\prime }$ to ${\text{SR}}_{\text{n}}^{\prime }$) fall into the detection range centered on start and end, length of which are 2L, respectively. Based on the extracted SRs and SR's, the boundary of the candidate CNV (blue area) is corrected in the expanded CNV (Blue area plus two gray areas). Next, the SRs that contain a substantial amount of breakpoint information are used to accurately define the boundaries of the mutated regions. According to the type of mutation zone, the correction procedure is composed of two parts. (1) The first part of the correction procedure is the correction of the candidate gain regions. The SM-type SRs are extracted in the gain region, which must meet the following conditions: a number of SRs greater than or equal to two, and a length of *S* that is the largest, which is perfectly matched in the expanded gain region. The SM-type SRs are used to calculate the starting point of the mutation region (*GREG*_*s*_) and the endpoint of the mutation region (*GREG*_*e*_), which are expressed by Equations (15, 16), respectively:

(15) $$\begin{array}{l} GREG_{s}=POS_{M}, \end{array}$$

(16) $$\begin{array}{l} GREG_{e}=POS_{S}+n-1, \end{array}$$

where *POS*_*M*_ indicates the alignment position of the first base at the left end of *M*, *POS*_*S*_ indicates the alignment position of the first base at the left end of *S*, which can be calculated using the string-matching method, and *n* indicates the length of *S*. An example of the boundary correction process of the CNV gain area is described in detail in Figure 3. An SR is split into two parts (M and S) from the breakpoint. M and S are compared to the expanded gain (red area), and their length (m and n) and position (POS_M_ and POS_S_) are used to correct the boundary of the expanded CNV (GREG_s_ and GREG_e_). (2) The second part of the correction procedure is the correction of the candidate loss regions. The MS-type SRs and SM-type SRs are extracted in the candidate loss regions. If the overlap between a MS-type SR and a SM-type SR exceeds 60%, this pair of SRs is considered to contain the same breakpoints. Moreover, the number of pairs of SRs is guaranteed to be more than two, and those SRs with the greatest length of *S* are chosen. A dynamic programming algorithm is adopted to calculate the overlapping ratio. MS-type SRs are used to calculate the starting point of the mutation region (*LREG*_*s*_), and SM-type SRs are used to calculate the endpoint of the mutation region (*LREG*_*e*_) (Wu et al., 2013), which are respectively described by Equations (17, 18):

(17) $$\begin{array}{l} LREG_{s}=POS+m, \end{array}$$

(18) $$\begin{array}{l} LREG_{e}=POS^{\prime }-1, \end{array}$$

where POS indicates the alignment position of the first base at the left end of *M* of the MS-type SR, *m* indicates the length of *M*, and *POS*′ indicates the alignment position of the first base at the left end of *M* of the MS-type SR. An example of the boundary correction process of the CNV loss area is described in detail in Figure 4. SR and SR' are split into two parts (M and S) from the breakpoint, respectively. The Ms and Ss are compared to the expanded loss (blue area), and their length (m, m', n, and n') and position (POS and POS') are used to correct the boundary of the expanded CNV (LREG_s_ and LREG_e_).

## Results and Discussion

To evaluate and verify the performance of RKDOSCNV, it was tested using both simulated and real data. In the simulated data experiment, the proposed method was evaluated by comparing it with four published algorithms (CNV_IFTV, CNVnator, FREEC, and SeqCNV). Three performance indicators (recall, precision, and F1-score) were adopted to assess the performance of each method. In the real data experiment, three types of data (three real samples from the 1,000 Genomes Project, two ovarian cancer samples and one breast cancer sample) were used to test RKDOSCNV. The CNVs of three real samples from the 1000 Genomes Project were provided in the Database of Genomic Variants, which was used as the ground truth file to calculate the recall, precision, and F1-score of each method to evaluate their performances. Via the analysis of the real data samples, it was found that RKDOSCNV detected that some CNVs have important biological significance, which can provide powerful assistance for cancer prevention and targeted drug development.

### Simulated Data Experiments

The simulated data sets were generated by IntSIM software (Yuan et al., 2017). Before using the software, the following settings were made for the reference genome as input, tumor purity (TP), and sequencing coverage (SC). Chromosome 21 of hg19 was chosen as the reference genome, the TP was set to 0.1 or 0.2, and the SC was set to 30×. The ground truth file was composed of six gains, four hemizygous losses (hemi-losses) and four homozygous losses (homo-losses). Under each set of settings, 50 simulation samples were generated.

**Figure 5:**
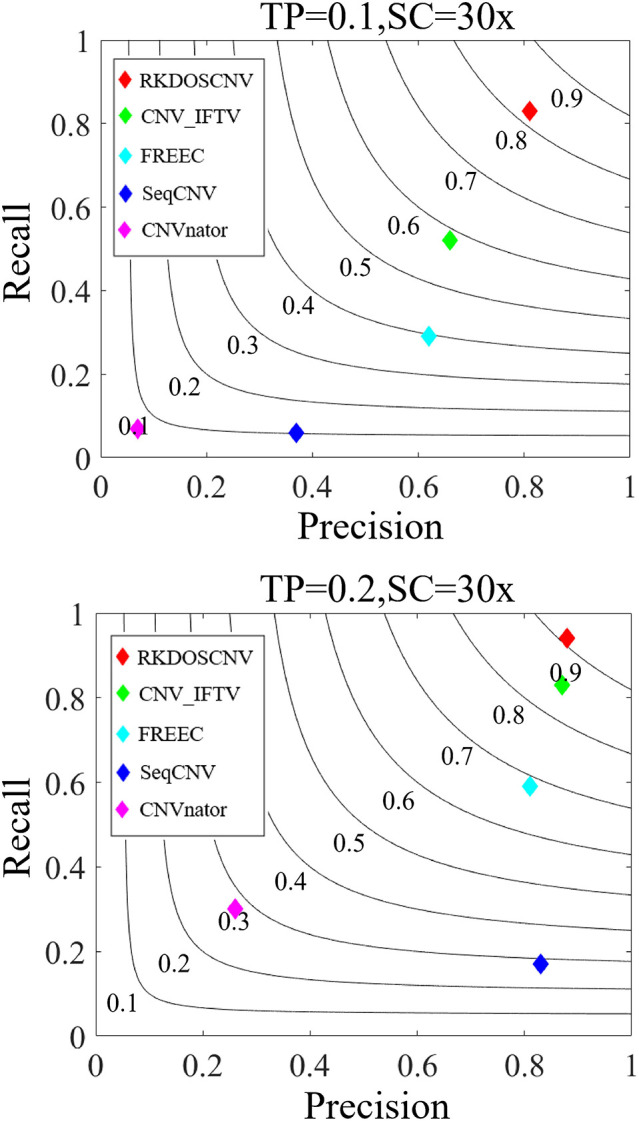
The performance of five methods was evaluated on two sets of simulated data sets. The F1-score of each method was calculated as the evaluation indicator of performance. Black curves indicate F1-score levels that are harmonic means of recall and precision. The equations on the left and right sides of the comma represent the tumor purity (TP) and sequencing coverage (SC), respectively.

Based on these simulated data sets, the performances of RKDOSCNV and four published methods (CNV_IFTV, CNVnator, FREEC, and SeqCNV) were tested to calculate their recall, precision, and F1-score. Recall is defined as the ratio between the number of CNVs correctly detected by a method and the total number of CNVs in the ground truth file. Precision is defined as the ratio between the number of CNVs correctly detected by a method and the total number of CNVs detected by the method. The F1-score is defined as the harmonic mean of precision and recall. The comparison results are presented in detail in Figure 5, from which it is evident that the overall trend of performance changes of each method increased along with the increase in TP. For example, the smallest F1-score was close to 0.1 when the TP was set to 0.1, and the largest F1-score was >0.9 when the TP was set to 0.2. Under each set of conditions, RKDOSCNV achieved the best F1-score, followed by CNV_IFTV, FREEC, SeqCNV, and finally CNVnator. CNVnator achieved the lowest precision under each set of conditions; it detected many long CNVs, most of which were false positives. SeqCNV achieved the lowest recall but higher precision, which demonstrates that the detection of this method was more conservative than the other methods. The performance of FREEC was better than those of CNVnator and SeqCNV. Its performance improved by nearly 0.3 when the TP was increased from 0.1 to 0.2, which demonstrates that it is not sufficiently sensitive to detect samples with extremely low purity. CNV_IFTV achieved a better tradeoff between recall and precision than these three methods. Of all the tested methods, RKDOSCNV achieved both the highest recall and precision. Via the preceding analysis and discussion, it is proven that RKDOSCNV is an effective and reliable CNV detection method.

**Figure 6:**
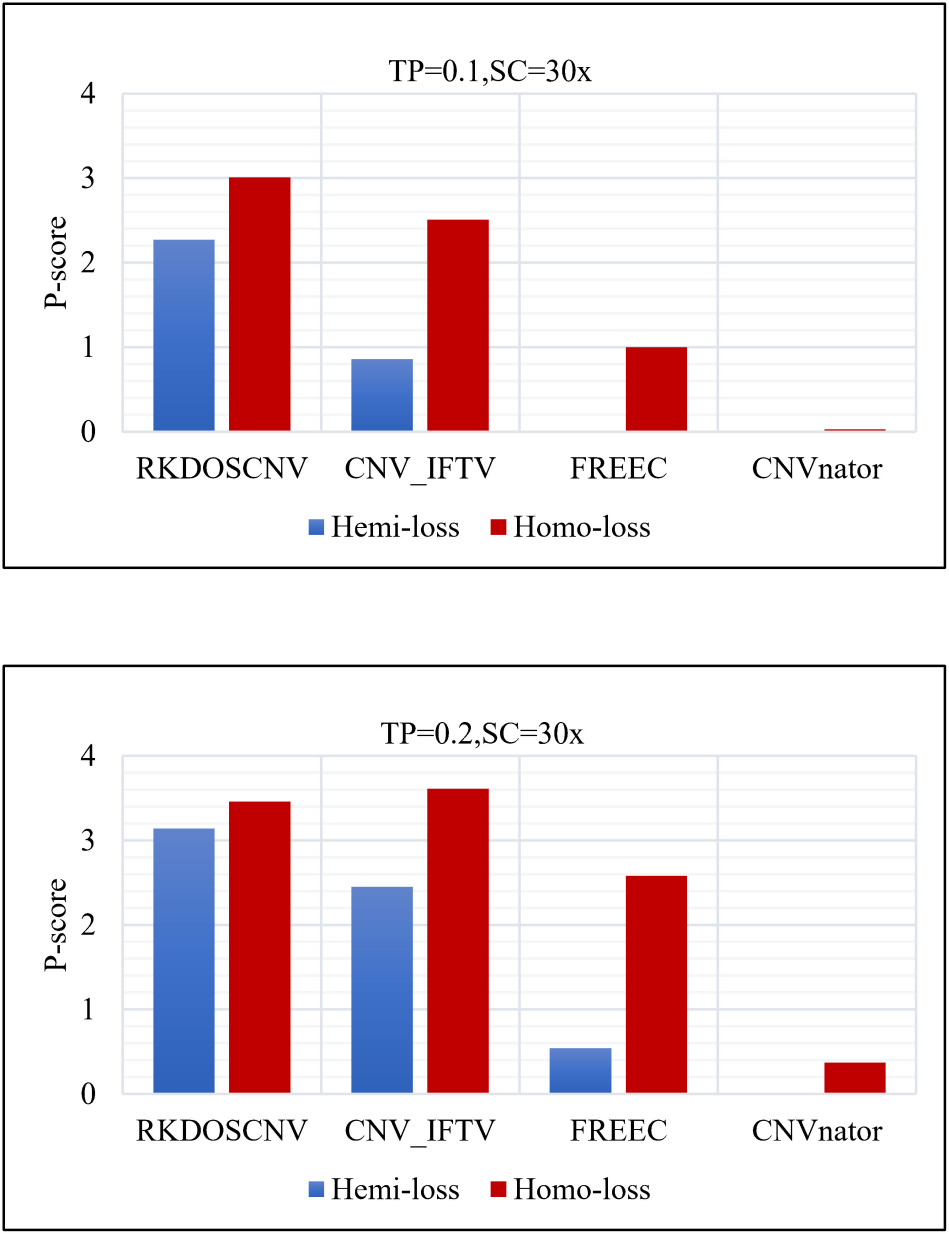
Histogram of the number of hemizygous loss (hemi-loss) and homozygous loss (homo-loss) for each method under two different sets of conditions. The equations on the left and right sides of the comma represent the tumor purity (TP) and sequencing coverage (SC), respectively.

To verify this conclusion, the detection power score (P-score) of each method was detected in the regions of insignificant variation (hemi-loss regions) where the copy number is one, which can easily be mistakenly detected as normal areas. The P-score is expressed as follows:

(19) $$\begin{array}{l} P-score=L_{n}\cdot \frac{L_{n}}{L_{n}+L_{fp}} \end{array}$$

where *L*_*n*_ represents the number of correctly detected loss areas (hemi-loss or homo-loss) and *L*_*fp*_ represents the number of false positive loss areas; the higher the P-score, the better the detection power. Moreover, the numbers of homo-loss regions were also counted. Four methods (RKDOSCNV, CNV_IFTV, CNVnator, and FREEC) were chosen for comparison, and SeqCNV was not included because it did not detect any loss areas. The P-scores of each method in both regions are described in detail in Figure 6, where it is evident that the P-score of each method presented an increasing trend with the increase of the TP from 0.1 to 0.2. The P-score of homo-loss of each method was higher than the P-score of hemi-loss of each method under all settings, which demonstrates that homo-loss can be more easily detected than hemi-loss. When the TP was equal to 0.1, the score of hemi-loss of RKDOSCNV was far superior to those of the other methods, which demonstrates that it is a reliable tool for the detection of insignificant CNVs. CNV_IFTV achieved higher P-scores and ranked second among all methods. FREEC achieved lower P-scores of hemi-losses than RKDOSCNV and CNV_IFTV, and did not detect any hemi-loss when the TP was equal to 0.1. CNVnator did not detect any hemi-loss under two sets of conditions. Overall, RKDOSCNV achieved the highest P-score, followed by CNV_IFTV, FREEC, and CNVnator, which is consistent with the conclusion of the simulation experiment.

**Figure 7:**
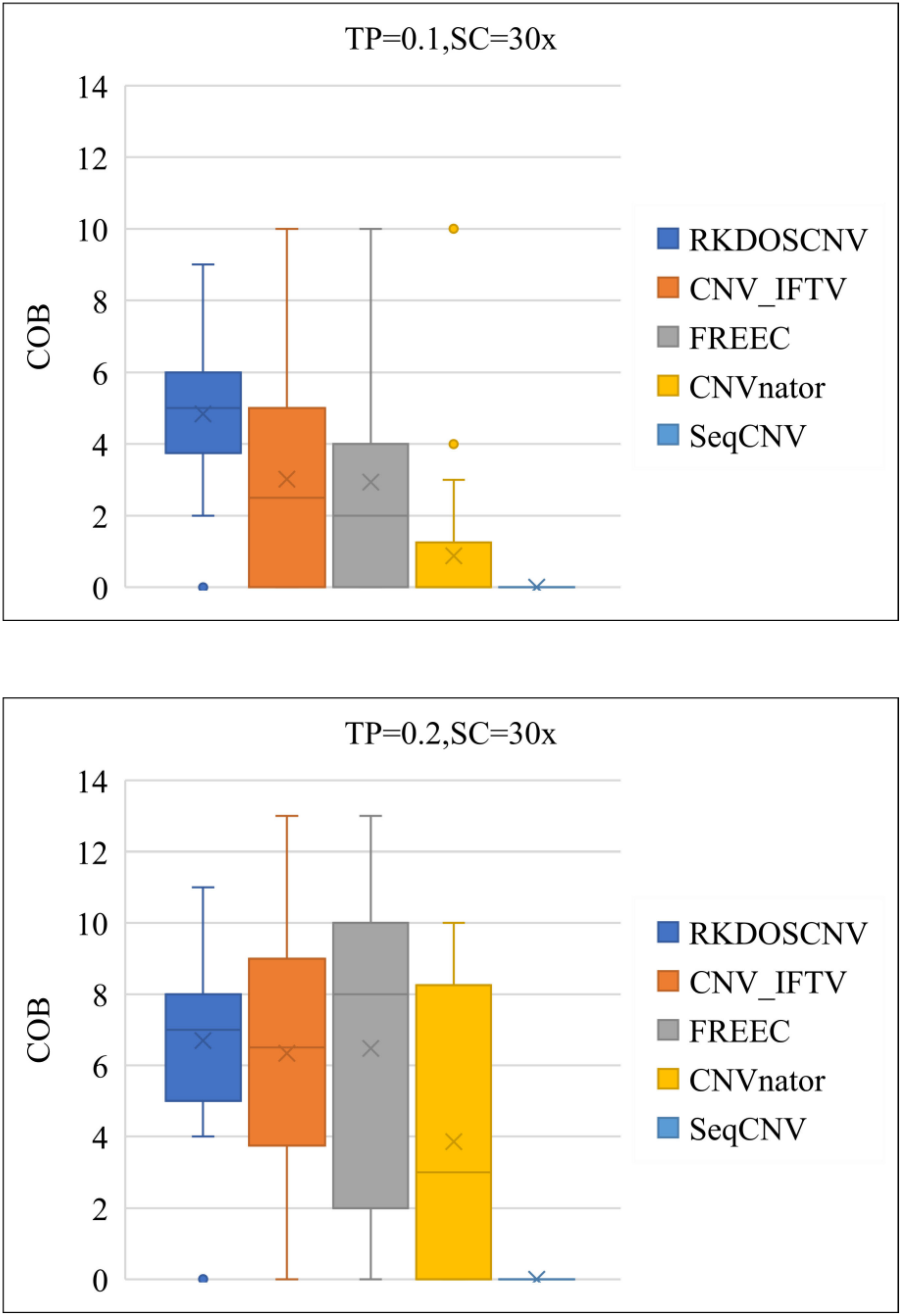
Box plots of the correctness of boundary (COB) of each method under two different sets of conditions.

As a supplement to these experiments, the correctness of the boundaries of correctly detected CNVs was further evaluated. The correctness of boundary (COB) is defined as the number of the boundary of correctly detected CNVs, the starting and ending points of which are consistent and compared with the ground truth file. As presented in Figure 7, that COB of each method is described using box plots under each set of simulation conditions, which includes 50 simulated samples. Figure 7 shows that the COBs exhibited an increasing trend with the increase of TP. RKDOSCNV achieved the highest average COB value under each set of conditions. The COB of CNV_IFTV was better than that of FREEC when TP was equal to 0.1; on the contrary, the COB of FREEC was better than that of CNV_IFTV when TP was equal to 0.2. Their COB values were relatively close under two sets of conditions. Both CNV_IFTV and FREEC get a higher COB than the other methods, but their average COB is lower than RKDOSCNV. CNVnator detected fewer correct CNV boundaries as compared to the above three methods, and SeqCNV could not detect the correct CNV boundaries under each set of conditions. Via the analysis and discussion of these experiments, it is evident that the performance of RKDOSCNV was the best among all compared methods, which fully verifies that it is a reliable and effective CNV detection tool.

### Detection of Real Samples From the 1,000 Genomes Project

**Figure 8:**
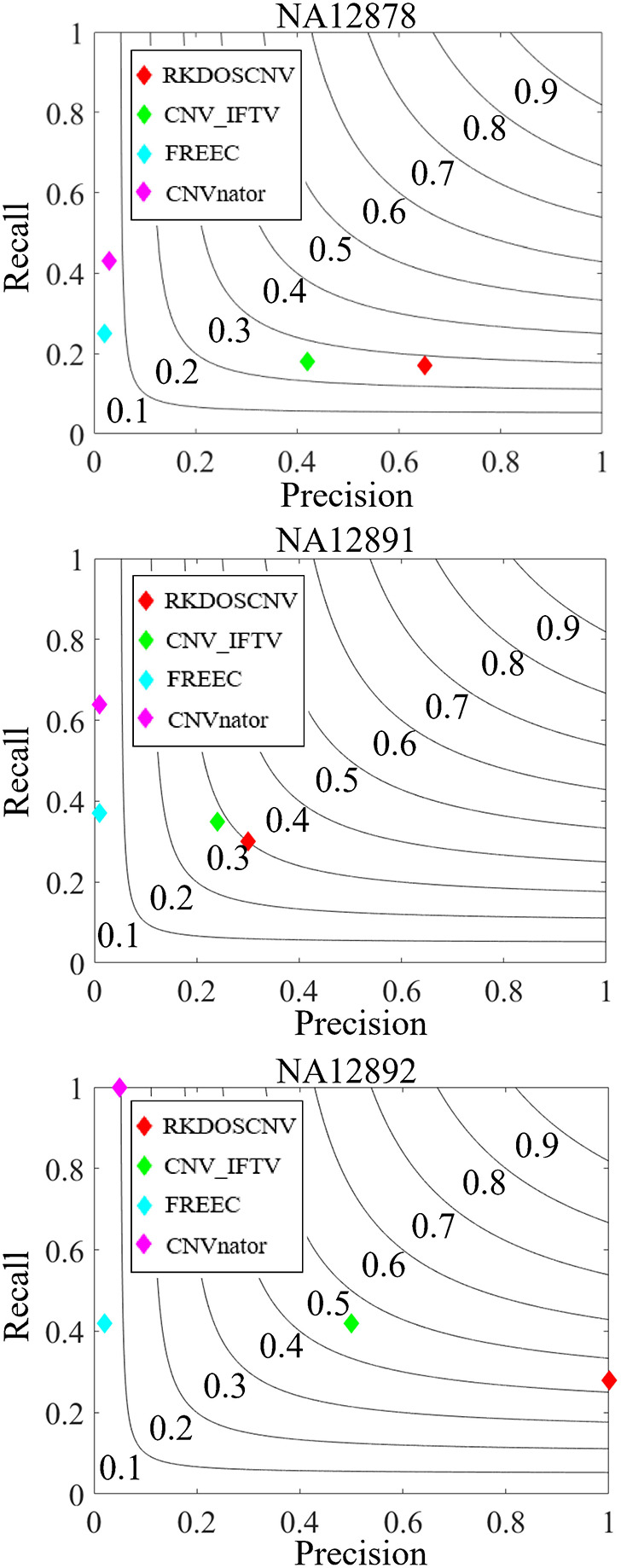
The performances of four methods were evaluated on three real data samples (NA12878, NA12891, and NA12892). Black curves indicate F1-score levels that are harmonic means of recall and precision.

To validate the proposed RKDOSCNV method, it was applied to the analysis of three real sequencing samples (NA12878, NA12891, and NA12892), which were downloaded from published article(Yuan et al., 2019). It was compared with three other single sample-based methods (CNV_IFTV, CNVnator, and FREEC). The test results of the three samples are recorded in the DGV database, by which the recall, precision, and F1-score of each method can be roughly calculated. The comparison results are described in detail in Figure 8. For the NA12878 sample, RKDOSCNV achieved the highest precision and a moderate recall. FREEC achieved a better recall and the lowest precision because it detected many false positives. CNV_IFTV achieved a better balance between recall and precision, and CNVnator achieved a lower precision and the highest recall. For the NA12878 sample, RKDOSCNV achieved the best F1-score, followed by CNV_IFTV, CNVnator, and FREEC. For the NA12891 sample, RKDOSCNV achieved the best F1-score, followed by CNV_IFTV, CNVnator, and FREEC. For the NA12892 sample, the F1-score of CNV_IFTV ranked first, followed by those of RKDOSCNV, CNVnator, and FREEC. Overall, from the results of the compared methods on the three samples, RKDOSCNV achieved a superior trade-off between recall and precision as compared to the other three methods. The preceding analysis and discussion demonstrate that RKDOSCNV is a relatively reliable CNV detection tool.

### Detection of Two Ovarian Cancer Samples

We used RKDOSCNV to detect the genome-wide data of two ovarian cancer samples (EGAR00001004838 and EGAR00001004839), which can download at published article (Yuan et al., 2018). Here, 22 autosomes of each sample are used for analysis. RKDOSCNV was compared with the other two methods (CNV_IFTV and FREEC). Number of overlapping events and Number of predicted events of each method are recorded in detail in Table 1. FREEC gets the most overlapping events, but it detects the most non-overlapping events. The number of overlapping events detected by RKDOSCNV is slightly less than FREEC. It detects a moderate number of non-overlapping events. CNV_IFTV Obtain the least overlapping events and non-overlapping events in each sample. In order to further compare the performance of each method, we use Equation (20) to calculate the overlapping density score (ODS) (Yuan et al., 2019) of each method.

(20) $$\begin{array}{l} ODS=M_{o}\cdot M_{p}, \end{array}$$

Where *M*_*o*_ represents the mean of the number of overlapping events (the mean of the intersection between one method and the other methods), *M*_*p*_ represents the ratio between *M*_*o*_ of the method and number of events predicted by it.

**Table 1:** Number of overlapping events and predicted events for each method on three real datasets.

**Sample ID**	**Type**	**RKDOSCNV**	**CNV_IFTV**	**FREEC**
EGAR00001004838	Number of overlapping events	10,912	2,319	11,529
	Number of predicted events	56,110	15,985	619,312
EGAR00001004839	Number of overlapping events	10,537	2,200	11,422
	Number of predicted events	22,764	17,308	625,767
PD4192a	Number of overlapping events	4,128	3,376	5,230
	Number of predicted events	16,672	13,848	306,257

The corresponding comparison results are listed in Table 2. RKDOSCNV get the highest ODS in each sample. In Table 1, FREEC detected the most overlapping events, but its ODSs are lower than RKDOSCNV. This is because it detects a large number of non-overlapping events. CNV_IFTV gets the lowest ODSs in two samples. In conclusion, RKDOSCNV predicted a moderate amount of CNV events in these samples and showed a relatively high overlapping density compared to other methods.

### Detection of a Breast Cancer Sample

**Figure 9:**
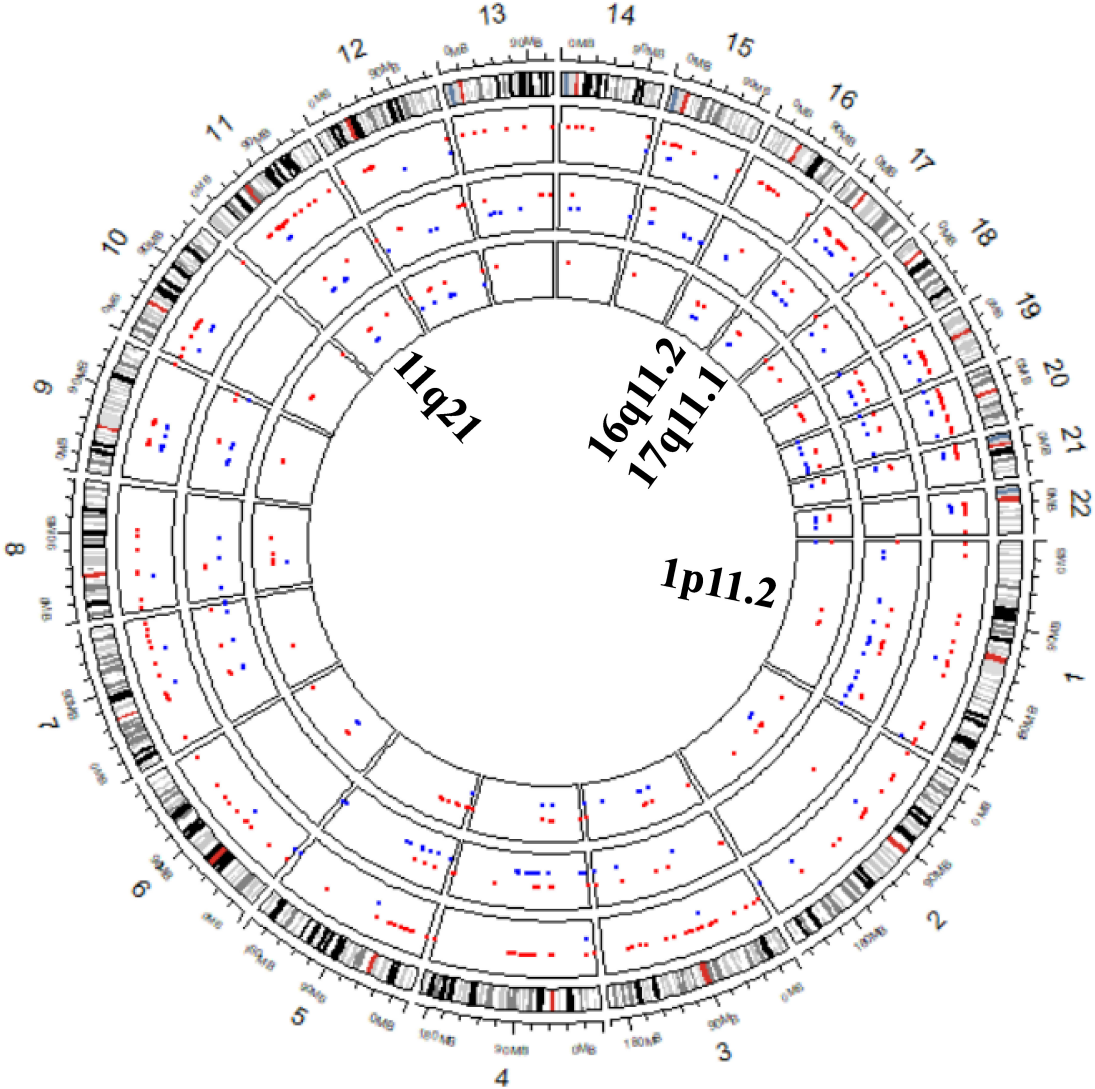
The Circos plot shows that the detected CNVs of each method are distributed in the 22 autosomes of a breast cancer sample. The red and blue dots represent gain areas and loss areas, respectively. The outermost ring describes the distribution of 22 autosomes. From the outside to the inside, the second, third, and fourth rings present the test results of FREEC, CNV_IFTV, and RKDOSCNV, respectively. The innermost ring corresponds to the four cytobands (1p11.2, 11q21, 16q11.2, and 17q11.1) detected by each method, which are associated with breast cancer.

To further validate RKDOSCNV, it was applied to the detection of CNVs in a breast cancer whole-genome sample (PD4192a), which was downloaded from published article (Li et al., 2019). The 22 autosomes of the breast cancer sample were extracted by SAMtools (Li et al., 2009). CNV_IFTV and FREEC were selected for comparison with RKDOSCNV. The experimental results were described in detail in Tables 1, 2. FREEC predicts the most CNV events and overlapping events, but it gets lowest ODS. It detected a large number of CNV events, most of which were long CNVs and proved to be false positives in the previous experiments. CNV_IFTV detects the fewest CNV events and overlapping events, and gets ODS between FREEC and RKDOSCNV. RKDOSCNV predicts the moderate number of CNV events and overlapping events, and it gets the highest ODS in all methods. Figure 9 presents an overview of the detected CNV distribution of the 22 autosomes, which is composed of four rings. From the outside to the inside, the first ring represents the distribution and length of the 22 autosomes, and the second, third, and fourth rings represent the detection of the gain and loss regions by FREEC, CNV_IFTV, and RKDOSCNV, respectively. The red and blue dots represent gain areas and loss areas, respectively. It was found that FREEC detects a large number of CNVs in each chromosome. CNV_IFTV did not detect any CNVs in chromosomes 6, 10, and 22, whereas the other two methods did detect CNVs. It shows that the detection of this method is more conservative than the other two methods. RKDOSCNV detected a moderate number of CNVs, and relatively more gain areas than loss areas.

**Table 2:** Summary of ODS for each method on three real datasets.

**Sample ID**	**RKDOSCNV**	**CNV_IFTV**	**FREEC**
EGAR00001004838	4,874	441	1,155
EGAR00001004839	7,177	432	1,982
PD4192a	1,739	1,279	725

Based on the preceding analysis, the biological meanings of detected CNVs were further investigated, many of which are associated with cancer or complex diseases. For example, the CNV gains at 1p11.2 (Jiang et al., 2011), 11q21 (Kazantseva et al., 2016), 16q11.2 (Savelyeva et al., 1994), and 17q11.1 are associated with breast cancer and are detected by each method, which is described in Figure 9. Those at 14q11.2 (Kawasaki et al., 2007) and 16p11.2 (Weiss et al., 2008) are respectively related to lung cancer and autism, that at 10q11.21 (Rees et al., 2016) is associated with schizophrenia, those at 1q21 (Grzasko et al., 2012) and 2q12.3 (Erickson et al., 2014) are associated with multiple myeloma, and those at 14q11.1 (Thean et al., 2018) and 18q21.1 (Druliner et al., 2018) are associated with colorectal cancer. CNV loss at 20q13.2 (Hidaka et al., 2000) is associated with colorectal cancer, that at 14q32.33 (Ledet et al., 2013) is associated with prostate cancer, that at 4q13.2 (Yang et al., 2008) is associated with osteoporosis, and that at 3q29 (Biamino et al., 2016) is related to autism. Via the preceding analysis, it is found that the four methods are very effective and can detect some valuable CNVs, and can therefore provide great assistance for clinical treatment and drug development.

## Discussion and Conclusion

In this study, a new method called RKDOSCNV was presented for CNV detection via the use of NGS data. RKDOSCNV was developed based on DOC and SR methods and uses a local perspective to detect CNVs, which replaces general methods that use global modeling to predict CNVs. RKDOSCNV is a single sample-based CNV detection method, and does not require the provision of paired samples. Via the verification of experiments on simulated and real data, it was proven that RKDOSCNV can detect many meaningful CNVs, and can provide effective assistance for the development of targeted drugs and cancer prediction. It is unique as compared to traditional detection methods that build statistical models based on global data and use hypothesis testing methods to predict CNVs. The three characteristics of RKDOSCNV are defined as follows. (1) Unlike traditional methods, RKDOSCNV does not need to make assumptions about the distribution of RD signals, and discerns the difference of RD signals from a local perspective. (2) By calculating the three types of neighbor relations of RDS signals, RKDOSCNV can successfully detect many insignificant CNVs. (3) Based on detected candidate CNVs, RKDOSCNV uses the SR approach to further determine the boundaries of candidate CNVs.

The effectiveness of RKDOSCNV was verified using both simulated and real data sets. In the simulated data experiment, RKDOSCNV was compared with four existing algorithms, and three performance indicators (recall, precision, and F1-score) of each method were analyzed to measure their performances. The ability of each method to detect insignificant CNVs and correctly identify the number of CNV boundaries was further evaluated. The experimental results demonstrate that RKDOSCNV achieved the best performance in terms of the F1-score, P-score, and COB. In the real data experiments, the performance of RKDOSCNV was evaluated using six real samples, and the biological significance of the detected CNVs was analyzed and discussed. Overall, RKDOSCNV is an effective and reliable CNV detection tool, especially for tumor samples of low purity.

During the experiment, some shortcomings were discovered. For example, the selection of the number of neighbors (*k*) is a critical step in the proposed method; it is set as an empirical value with reference to the traditional outlier detection methods (Breunig et al., 2000; Jin et al., 2006; Tang and He, 2017), which meets the needs of most situations, but may not be suitable in extreme cases. The threshold (θ) setting also has a great influence on the accuracy of the detection results. Based on applications in different scenarios (Tang and He, 2017), a moderate baseline was chosen to meet the application needs. The proposed method does not support detection of interspersed amplification, which is an important form of mutation. We will expand the functionality of the method so that it can detect multiple types of mutations. The CNVs detected by RKDOSCNV includes germline and somatic CNVs, but it cannot distinguish between the two variant types. If a control sample is input, our method can identify germline and somatic CNVs. Currently, there is no matched control sample, so the detection result is a mixture of two types of variants. In this study, the methods compared by RKDOSCNV detect all CNVs (germline and somatic CNVs). In future work, these problems will be addressed to improve the performance of RKDOSCNV, and reasonable methods will be developed to automatically select the optimal parameters and effectively identify other types of mutations. Based on the existing methods, other distance measurement methods and density evaluation methods will be chosen to further improve the performance of RKDOSCNV in the accurate and effective identification of CNVs.

## Data Availability Statement

The datasets presented in this study can be found in online repositories. The names of the repository/repositories and accession number(s) can be found in the article/Supplementary Material.

## Author Contributions

GL participated in the design of the algorithms and experiments. JZ and XY participated in the design of the entire framework of CNV detection and directed the whole work. CW participated in the analysis of the performance of the proposed method. JZ and XY conceived of the study and helped revise the manuscript. All authors read the final manuscript and agreed on its contents for submission.

## Conflict of Interest

The authors declare that the research was conducted in the absence of any commercial or financial relationships that could be construed as a potential conflict of interest.
