# Supplementary material for: RKDOSCNV: A Local Kernel Density-Based Approach to the Detection of Copy Number Variations by Using Next-Generation Sequencing Data
Source: Front Genet. 2020 Nov 4;11:569227. doi: 10.3389/fgene.2020.569227 (PMC7673372; doi:10.3389/fgene.2020.569227)
Supplement: Supplementary file 1 [file Data_Sheet_1.docx]

Supplementary Material

# Supplementary Tables

**Supplementary Table 1.** Sensitivity analysis of binsize on 50 simulation samples.
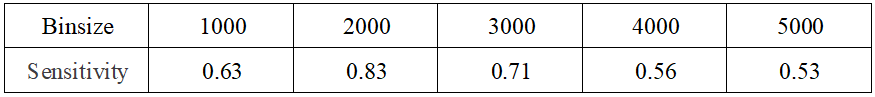


**Supplementary Table 2.** Sensitivity analysis of parameter k on 50 simulation samples.
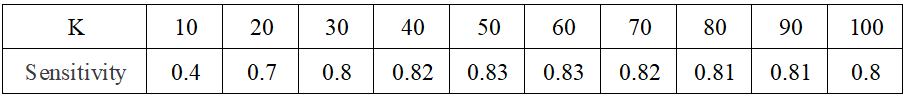


**Supplementary Table 3.** Sensitivity analysis of threshold (θ) on 50 simulation samples.
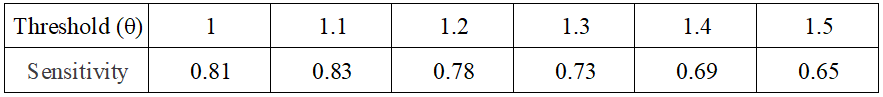


**Supplementary Table 4.** Sensitivity analysis of depth of exploration (m) on 50 simulation samples.
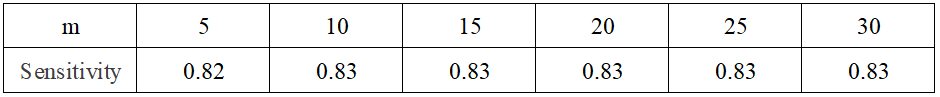


**Supplementary Table 5.** Summary of F1-scores of five methods based on simulation data.
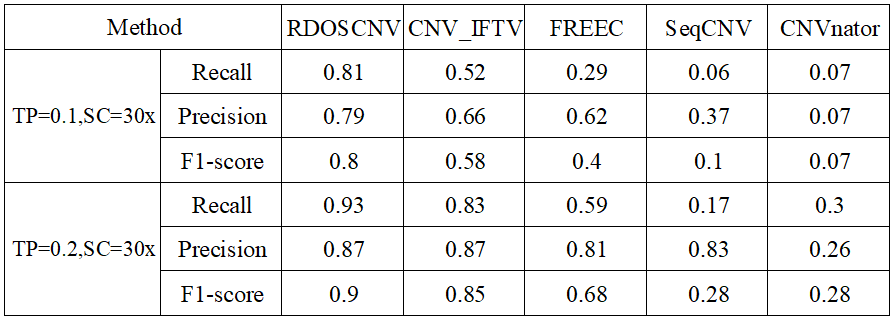


The equations on the left and right sides of the comma represent the tumor purity (TP) and sequencing coverage (SC), respectively. Here, each method only uses RD information to predict CNVs.
